# Carbon Monoxide and Nitric Oxide as Examples of the Youngest Class of Transmitters

**DOI:** 10.3390/ijms22116029

**Published:** 2021-06-02

**Authors:** Alicja Nowaczyk, Magdalena Kowalska, Jacek Nowaczyk, Grzegorz Grześk

**Affiliations:** 1 Department of Organic Chemistry, Faculty of Pharmacy, Ludwik Rydygier Collegium Medicum in Bydgoszcz, Nicolaus Copernicus University in Toruń, 2 dr. A. Jurasza St., 85-094 Bydgoszcz, Poland; magda.kowalska@doktorant.umk.pl; 2 Department of Physical Chemistry and Physicochemistry of Polymers, Faculty of Chemistry, Nicolaus Copernicus University, 7 Gagarina St., 87-100 Toruń, Poland; jacek.nowaczyk@umk.pl; 3 Department of Cardiology and Clinical Pharmacology, Faculty of Health Sciences, Ludwik Rydygier Collegium Medicum in Bydgoszcz, Nicolaus Copernicus University, 75 Ujejskiego St., 85-168 Bydgoszcz, Poland; g.grzesk@cm.umk.pl

**Keywords:** carbon monoxide (CO), nitric oxide (NO), neurotransmitters

## Abstract

The year 2021 is the 100th anniversary of the confirmation of the neurotransmission phenomenon by Otto Loewi. Over the course of the hundred years, about 100 neurotransmitters belonging to many chemical groups have been discovered. In order to celebrate the 100th anniversary of the confirmation of neurotransmitters, we present an overview of the first two endogenous gaseous transmitters i.e., nitric oxide, and carbon monoxide, which are often termed as gasotransmitters.

## 1. Introduction

The year 2021 is the 100th anniversary of the confirmation of the neurotransmission phenomenon by Otto Loewi [1,2]. The second half of the 20th century was the golden era for the identification of the major neurotransmitters in the mammalian central nervous system (CNS) [3].

Neurotransmitters are chemicals that enable communication, i.e., the flow of nerve impulses between nerve cells, or between nerve cells and muscles and glands. Recently, one can distinguish excitatory and inhibitory mediators, which are endo– or exogenous compounds that control the function of the entire organism. From a chemical point of view, neurotransmitters belong to many different structural groups including amino acids (such as glycine), peptides (such as substance P, somatostatin), monoamines (such as noradrenaline or dopamine), purine derivatives (such as adenosine), gases (such as nitric oxide, NO, carbon monoxide CO), and acetylcholine. From a medical point of view, disturbances in the concentration of neurotransmitters in the body result in the occurrence of mental disorders and diseases (such as depression, schizophrenia, Parkinson’s disease), which contribute to the occurrence of dementia (including Alzheimer’s disease) and others. For this reason, they are used in medicine (e.g., as antidepressants), but they can also be a serious problem in non-medical use (e.g., as a psychoactive substance such as rape pills).

NO and CO are the first two endogenous gaseous transmitters identified and are often termed as gasotransmitters [4]. NO was proclaimed the “Molecule of the Year” in 1992. The 1998 Nobel Prize in Physiology or Medicine was awarded for the discovery of nitric oxide’s role as a cardiovascular signaling molecule [5,6].

NO is an important cellular signaling molecule that participates in diverse physiological functions in mammals, including vasodilation, smooth muscle relaxation, neurotransmission [3,7], and the immune response [8,9,10,11].

The second gas transmitter, CO, is a product of heme catabolism, and is usually regarded as a toxic species that disrupts cellular respiration. However, recently, CO has been found to be an important signaling molecule and protector of tissues against injuries induced by several types of stress [12].

The purpose of this review is focused on description of the gasotransmitters from various perspectives. In the paper, the basic chemical and biological properties of CO and NO are addressed to provide a proper foundation for further discussion. Selected pharmaceutical data are presented later in the text.

## 2. CO and NO—Chemistry

Both CO and NO are diatomic gaseous oxides of carbon or nitrogen, respectively. CO is a colorless, odorless, and tasteless flammable gas that is slightly less dense than air [13]. NO is one of the principal oxides of nitrogen and also is a colorless gas [5]. Spectroscopic data are available in the literature and databases: Fourier Transform Infrared (SpectraBase Spectrum ID: KlmI6BZQQsa [14] and GeuwXWi3T6m [15] for CO and NO, respectively) UV/VIS [16] (CAS RN: 630-08-0 for CO [17] and 10102-43-9 for NO [18]) GCMS (accession: NIST MS number 19 for CO [19] and NIST MS number 31 for NO [20]).

**Figure 1:**
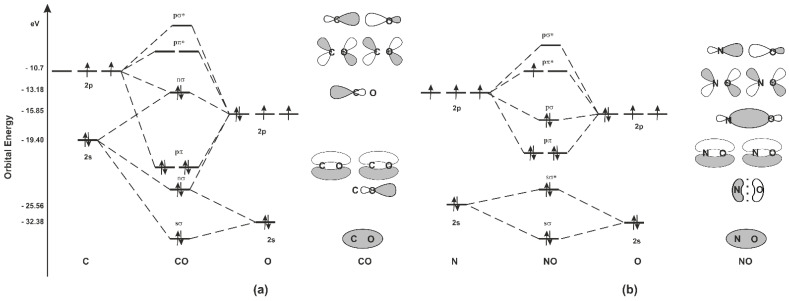
Quantitative energy level and molecular orbital diagram of CO (**a**) and NO (**b**) with electronic occupancy in the ground state. The asterisk identifies anti-bonding orbitals. Schematic shape of the bonding and frontier molecular orbitals of CO and NO. Black and white colors represent the phases of the orbital lobes.

CO is a stable, naturally occurring compound with carbon in a 2+ oxidation state. The molecule has ten valence electrons, distributed among three bonds and one lone pair on each atom (Figure 1) [21]. NO has one non-paired electron, so being a free radical has a high reactivity. The NO molecule is lipophilic [22].

To better understand how CO and NO bind to transition metals, it is necessary to understand the bonding of CO and NO. The carbon, nitrogen, and oxygen atoms possess one 2s and three 2p orbitals. The carbon, nitrogen, and oxygen lay next to each other in the periodic table of elements and their valence shell consists of 2s and 2p orbitals. The carbon atom has four valence electrons distributed in that shell, while nitrogen has five and oxygen six valence electrons (Figure 1). As depicted in Figure 1, CO molecular orbitals in the ground state are occupied following molecular orbitals: one σ-bonding orbitals, two π-bonding orbitals, two nonbonding orbitals (frequently referred to as lone pairs), and one σ-antibonding orbital. In this case, there are three bonding orbitals occupied and no antibonding orbitals occupied, giving a bond order of three. Due to this, CO is a very stable 10-valence electron molecule, isoelectronic with CN^−^, NO^+^ and with N_2_. Occupied nonbonding orbitals on the carbon and on the oxygen point away from the molecule and can interact with transition metals (M) via covalent (coordinational) bonds [23]. This means that the major component in giving the bond strength is the delocalization of the bonding electrons through the interaction between the M and CO orbitals. The HOMO of CO (nσ) donates its electron pair to an empty metal orbital forming a σ bond. This σ-bond alone is not strong enough to hold the CO ligand to the metal but it is strengthened by π-backbonding, leading to stabilization of the system. The formation of the second is possible because the LUMO orbitals of CO (pπ*) have adequate symmetry to overlap with filled d orbitals of the metal and form an additional molecular orbital π referred to as π-backbonding [21]. CO having two unoccupied π-antibonding orbitals is an acceptor of electrons and is called a π acceptor or π acid. CN^–^ and NO^+^ are also biologically relevant π acceptors. A good example of the reactivity control provided by M–CO backdonation and the unique binding characteristics of CO is shown by hemoglobin (Hb) [24], which binds CO, forming carboxyhemoglobin (COHb) when heme is reduced (Fe^2+^), and releases it upon oxidation to methemoglobin (metHb) (Fe^3+^). CO, like NO, avidly binds Fe(II)Hb, but unlike NO does not bind Fe(III)Hb.

In the case of NO, the two lowest molecular orbitals (sσ, sσ*) (Figure 1) can basically be considered nonbonding orbitals because they do not contribute to the reactivity of the molecule. The more energetic antibonding pσ* is identified as the nitrogen nonbonding electron pair. The chemical properties of NO molecules are strongly influenced by the σ nature (symmetry, energy) of this nonbonding pσ* molecular orbital. The remaining unpaired electron occupies the pπ* antibonding orbital, polarizing the NO molecule. This situation leads to a half-filled HOMO orbital, which is characteristic for radicals. According to this in the literature, the HOMO orbital of radical is given a special name: a single occupied molecular orbital (SOMO) [25]. The SOMO is used in radical recombination reactions and represents the orbital in which an electron is removed or added in redox reactions. Therefore, the nature, symmetry, and particularly the energy of the SOMO are critical to radical reactivity. NO is a poor oxidant and a poor reducing agent under physiological conditions [26]. In addition, NO is an uncharged molecule and too weak of a Lewis base to act as a nucleophilic agent. Spin pairing with other species containing unpaired electrons, such as other radicals and transition metal ions, provides the only known rapid reactions of NO with biological compounds/intermediates [27]. Therefore, despite being a free radical, NO reacts with only a few targets in cells, and this preference or selectivity was very important for the evolution of NO as a biological mediator [28]. The nitrogen is polarized in the pπ*, which explains why recombination reactions with transition metal ions and radicals take place exclusively through the nitrogen atom. This polarization also explains the selectivity of NO reactions [25].

Nitrosylation is the basic and most biologically important reaction of NO [26]. NO is a common ligand for metals since its reactivity is a consequence of the favorable symmetry and energetic interactions of metal d orbitals and NO orbitals [28]. This interaction results in multiple, strong M-NO bonds. The affinity of NO for Fe(II) in Hb is between 8000- [29] and 1500-fold [30] greater than that of CO, while the affinity of Hb for CO is approx. 220 times greater than O_2_ [31]. The Fe−N−O bond shape in nitrosyl species varies from a linear in the case of the Fe^2+^-NO^+^ to a bent unit in the case of the formal oxidation state Fe-NO^−^, and the intermediate shape for Fe−NO [27]. The studies of formal charge on NO indicate that bound NO behaves as an electrophile or a nucleophile [25].

Table 1 compares the physical properties of discussed gasotransmitters. From a chemical point of view, CO is the most biologically stable gasotransmitter out of known three biologically important gases (NO/CO/H_2_S) [32]. CO has weak chemical reactivity, mainly because it does not have unpaired electrons, and does not dissociate in an aqueous solution to form different chemical species (Figure 1, Table 1). According to this, CO may be capable of exerting its effects during longer time periods and distances compared to NO [33].

## 3. CO and NO—Endogenous Production

The discovery that mammalian cells produce both CO and NO provides important information on many biological processes. The fact is even more intriguing, taking into account that they are listed among highly toxic air pollutants. Of course, there is a significant difference in the concentration between these two cases. In the living organism, CO and NO occur at very low concentration.

**Figure 2:**
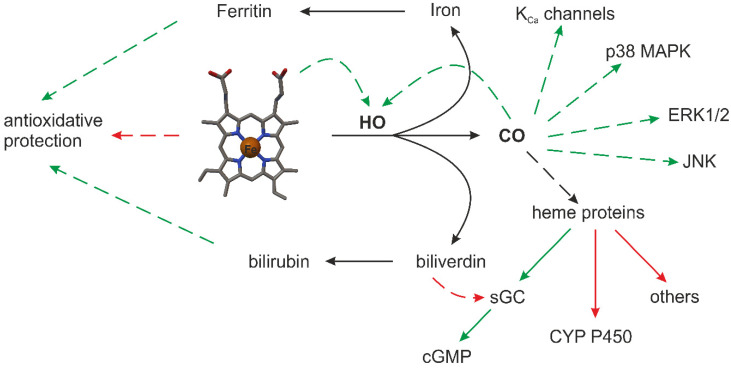
Schematic illustration of endogenous CO synthesis by heme oxygenase (HO) and the subsequent cyclic guanosine monophosphate (cGMP) signaling cascade reaction [32]. Solid lines represent metabolic routes or subgroupings. Dashed lines represent main functional consequences: stimulation (green line); inhibition (red line). Exceptions exist for the depicted functional consequences. Soluble guanylate cyclase (sGC), calcium signaled potassium channels (KCa), and p38 mitogen activated protein kinases (p38 MAPK) together with inhibitors cytochrome P450 (CYP450), c-Jun N-terminal kinase (JNK) and the extracellular signal-regulated kinase (ERK1/2) pathway.

The endogenous production of CO is a continuous process of heme degradation (i.e., catabolism of heme) in mammalian cells (Figure 2) [33].

**Figure 3:**
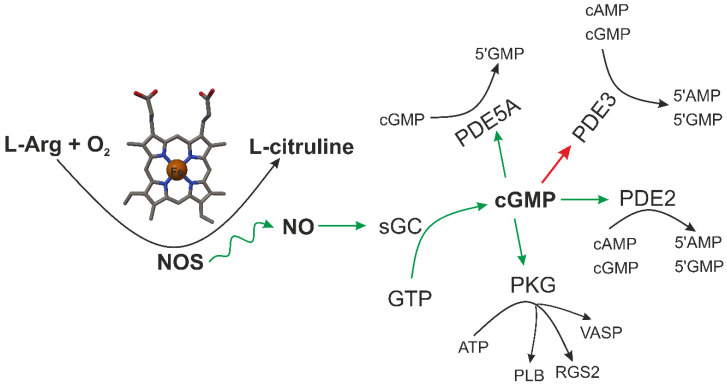
Schematic illustration of endogenous NO synthesis by NOS [45]. and subsequent cyclic guanosine monophosphate (cGMP) signaling cascade reaction [46]. Upon nitric oxide synthase (NOS) activation soluble guanylate cyclase (sGC) from guanosine triphosphate (GTP) cGMP is produced. cGMP can then activate cGMP-dependent protein kinase (PKG) and either activate (green line) or inhibit (red line) various phosphodiesterase (PDE) isoforms. PKG phosphorylates several protein targets, including phospholamban (PLB), vasodilatory-stimulated phosphoprotein (VASP), regulator of G protein signaling 2 (RGS2). PDE2 and PDE3 catabolize both cyclic adenosine monophosphate (cAMP) and cGMP, whereas PDE5 specifically catabolizes cGMP. Upon cGMP binding to its regulatory GAF domain, PDE2 undergoes a conformational change and increases its enzymatic activity for cAMP. PDE5 similarly increases its catalytic activity for cGMP by an order of magnitude upon cGMP binding to its regulatory small-molecule-binding domains i.e., GAF domain (found in cGMP, cAMP, FhlA) [47,48].

NO synthesis is a complex multistep biological process [39]. NO is produced by various cells in the body and is mediated by a family of enzymes called nitric oxide synthases (NOSs, EC 1.14.13.39 [40]) by the oxidation of L-arginine (L-Arg) to L-citrulline (Figure 3). This requires cosubstrates such as L-Arg, nicotinamide adenine dinucleotide phosphate (NADPH), flavin adenine dinucleotide (FAD), and O_2_ along with cofactors such as calmodulin and tetrahydrobiopterin (BH_4_) [41,42]. There are three homologous NOS isoforms whose name is determined by the tissue from which they were originally isolated in the mammalian body [43]. Two of them, NOS1, i.e., neuronal NOS (also known as Type I, NOS I, and nNOS) and NOS3 i.e., endothelial NOS (also known as Type III, NOS III, and eNOS), are constitutively expressed, while the third one is inducible and is thus termed NOS2 i.e., inducible NOS (also known as Type II, NOS II, and iNOS) [44]. This multiple location of the synthesis suggests a variety of different regulatory roles for NO under physiological and pathophysiological conditions [41]. NOS1 is primarily found in the nervous system and is necessary for neuronal signaling, while NOS3 is present in the endothelium and plays an essential role in vasodilation and the control of blood pressure [8]. These two isoforms produce nanomolar (10^−9^ M) amounts of NO for short periods of time (seconds to minutes) in a calcium/calmodulin (CaM)-dependent manner [8]. NOS1 and NOS3 produce NO for predominantly signaling purposes (Figure 3).

Heme serves as a vital cofactor in oxygen transport proteins (hemoglobin, myoglobin) and in enzymes involved in critical cellular processes such as respiration, inflammation or drug metabolism. It occurs through the activity of two isoforms of enzymes called heme oxygenase (HO E.C. 1:14:99:3 [49]): constitutive (HO-2) and inducible (HO-1), which are products of two different genes, HMOX2 and HMOX1 [50]. Additionally, HO-1 is a heat shock protein (HPS) and is an essential antioxidant enzyme upregulated in response to cellular stress. HO-2 as a constitutively expressed enzyme is mainly responsible for primary HO activity [51]. CO is an important signalling mediator possessing vasodilatory properties, which are achieved by activation of the guanylate cyclase–cGMP pathway as well as non-cGMP pathways. Non-cGMP pathways for CO appears to modulate large-conductance potassium channels (K_Ca_) and p38 mitogen activated protein kinases (p38 MAPK) together with inhibitions CYP450, c-Jun N-terminal kinase (JNK) and the extracellular signal-regulated kinases (ERK1/2) pathway (Figure 2). The activity of these constitutively expressed enzymes is regulated by the Ca^2+^ concentration through binding of the Ca^2+^–calmodulin complex [52]. NOS2 was first isolated from macrophages and expressed only following induction by inflammatory mediators such as tumor necrosis factor α (TNFα, interferons type II (IFN-γ), interleukin family (IL family: IL-1, IL-1β, IL-2, IL-10), lipopolysaccharide/endotoxin (LPS) [53,54]. The activity of NOS2 appears to be independent of the Ca^2+^ concentration. The major role of NOS2 appears to be in host defense through the cytotoxic effects of high NO levels [55]. After induction, NOS2 generates significant amounts of NO (micromolar range 10^−6^ M), which persists until the enzyme is degraded, sometimes for hours. All three isoforms generate NO by oxidizing a guanidine-nitrogen group from L-Arg, utilizing NADPH as an electron donor [39,56]. It is estimated that NOS2 produces 20-fold more NO than NOS1 and NOS3 together.

**Figure 4:**
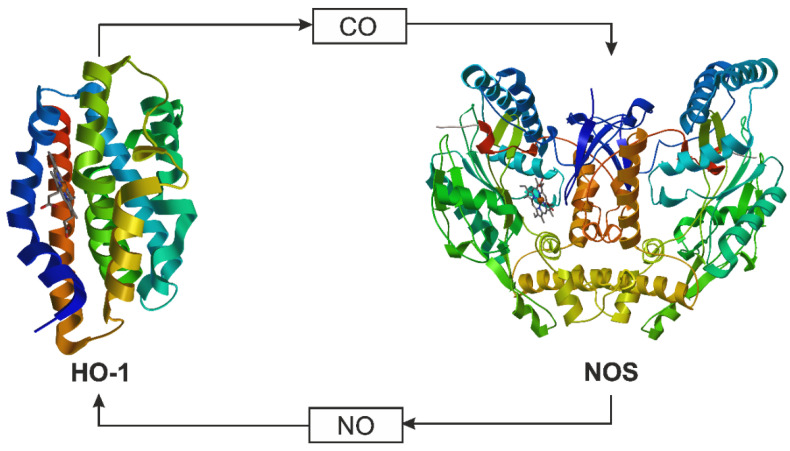
The metabolic gas cycle for CO and NO. The enzymes nitric oxide synthase (NOS: PDB ID: 4NOS, 2.25 Å [52]) and heme oxygenase 1 (HO-1: PDB ID: 4G98, 2.30 Å [58]) produce NO and CO, respectively, and NO and CO can enhance or inhibit enzyme activity, as dictated by cellular need [34].

Recent data show that NO and CO production are coupled in the metabolic gas cycle; NO generated by NOS increases HO-1 expression, increasing CO production (Figure 4) [33]. In other words, both can be a modulator of their signaling [32]. Additionally, some findings point to the possibility that HO-1 and/or CO and NOS2 and/or NO are functionally related to mediating their protective effects. In endotoxic shock, the salutary action of CO in rat brain appears to depend sequentially on the activation of nuclear factor kappa-light-chain-enhancer of activated B cells (NFkB), which triggers transcription of NOS2 with the production of NO, and subsequently the upregulation of HO-1 [57].

## 4. CO and NO—Biological Properties

Chemical Entities of Biological Interest under the CHEBI entry: 17,245 for CO [13] 16,480 for NO [5]) belong to the gasotransmitters group and according to the literature, they are the smallest and simplest of biological active molecules in nature. Comprehensive information about human CO and NO metabolites can be found in the Human Metabolome Database [59] under the acronym HMDB0001361 [60]; HMDB0003378 [61] and Kyoto Encyclopedia of Genes and Genomes [62] under the entry D09706 [63] and D00074 [64].

CO and NO as chemical compounds were discovered, similar to oxygen, in 1770 by Joseph Priestley [65]. Research into these molecules’ biochemistry began about 200 years later (approx. 1977). NO activates soluble guanylate cyclase (sGC), an enzyme that converts guanosine triphosphate (GTP) into cyclic guanosine monophosphate (cGMP), a secondary relay molecule known since 1963. This signal transduction activates various protein kinases such as protein kinase G (PKG) and others (Figure 3) [45].

NO is a small and diffusible free radical that acts as a secondary messenger throughout the human body [11]. NO is not only the end product of the NOS enzyme, but also newly generated NO can bind to heme as a feedback inhibitor [66]. It relaxes vascular smooth muscle by binding to the heme moiety of cytosolic GC, activating guanylate cyclase and increasing intracellular levels of cyclic guanosine 3′,5′-monophosphate, which then leads to vasodilation (Figure 3). This, in turn, regulates many significant biochemical pathways in the vascular system. When inhaled, NO produces pulmonary vasodilation [67]. Due to this, interaction of NO with the heme protein of sGC (Figure 3) is perhaps the most important/critical example of a direct biological effect of NO. NO can interact with a number of other heme proteins [68] including Hb, cyclooxygenase (COX), cytochrome P450, and cytochrome c oxidase [69]. Changes in the concentration of NO in the body reveal the dual nature of NO activity (beneficial/detrimental). High concentrations (due to, e.g., overexpression or dysregulation of NOS2) cause toxic effects associated with various human diseases such as septic shock, cardiac dysfunction, pain, diabetes, and cancer. Physiological concentrations of NO ensure its proper biological functions, including vasodilation [70], smooth muscle relaxation [71], platelet inhibition [72], neurotransmission [3,7], and immune response [9,73]. Numerous studies have confirmed that at high concentrations NO becomes cytotoxic, and antimicrobial, additionally it induces prooxidant response and apoptosis. For instance, high levels of NO (concentration 10^−6^ M) generated by NOS2 reacts with various reactive oxygen species to produce reactive nitrogen species, which exert indirect effects, such oxidation, nitration, and nitrosation. At low concentrations ranging from 10^−12^ to 10^−9^ [74], circulation of NO leads to protective effects such as reduced oxidative stress and angiogenesis [75]. For example, low levels of NO produced by NOS1 and NOS3 directly interact with specific molecules, such as metals, lipid radicals, and DNA radicals. NO has also broad effects on cancer, from cancer initiation of cellular transformation to cancer progression of the metastatic cascade [76]. Therefore, the regulation of NO production is important both to maintain its normal physiological functions and to control its harmful effects [45].

**Figure 5:**
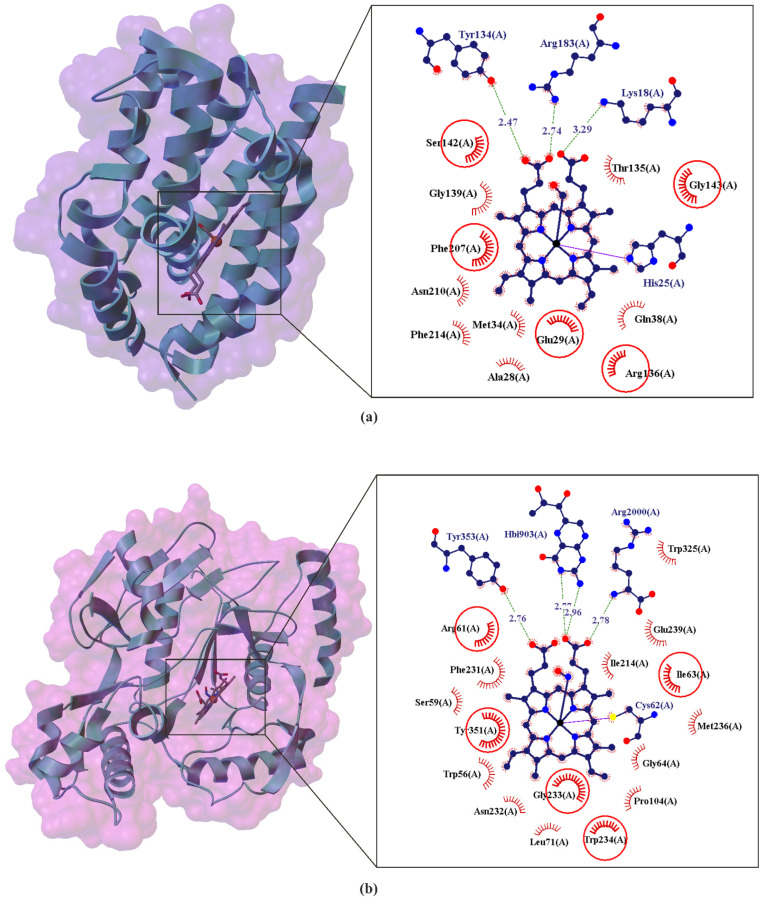
Comparison of the X-ray structure of Heme Oxygenase-1 (HO-1) binding carbon monoxide (CO) (**a**) and Nitric oxide synthase (NOS) binding nitric oxide (NO) (**b**) neurotransmitters in the heme region. The enlarged area shows the structural elements around the CO ligand binding site (PDB ID: 4G98, 2.30 Å) and NO ligand binding site (PDB ID: 2FC1, 2.00 Å) (**b**). Residues that form hydrogen bonds (dashed lines) with heme are shown in ball-and-stick representation with the interatomic distances shown in Å. Residues forming Van der Waals interactions with heme are shown as labeled arcs with radial spokes that point toward the ligand atoms. The red circles and ellipses identify equivalent residua (in 3D superposition of structures).

The toxicity of CO has been known since ancient Greek and Roman times. The mechanism of CO toxicity is not complicated since it does not create any toxins in the body. However, it does not poison the body with complex chemical compounds; CO works simply and effectively, displacing oxygen from the organs and blocking its transport. (Figure 5). Therefore, it is toxic to animals that use Hb as an oxygen carrier. Concentrations higher than 35 ppm trigger CO poisoning mechanisms [13]. CO exerts effects on cell metabolism through both hypoxic and non-hypoxic modes of action. Both mechanisms of action are thought to be the result of the ability of CO to bind strongly to heme and alter the function and/or metabolism of heme proteins. CO’s high diffusivity and lack of reactivity in cells and tissues permit it to access more cellular targets than NO, which is unable to act far from its generation source due to higher reactivity and thus a shorter life span (Table 2). The binding affinity of CO to Hb is more than 200 times greater than that of oxygen to Hb (Table 1) [29]. The formation of carboxyhemoglobin (COHb) (Figure 5) decreases the O_2_ carrying capacity of blood and disrupts the release of O_2_ from Hb for its use in tissues.

Through similar mechanisms, CO diminishes the O_2_ storage in muscle cells by binding to and displacing O_2_ from myoglobin. Though all human tissues are vulnerable to carbon monoxide-induced hypoxic injury, those with the highest O_2_ demand are especially vulnerable, including the brain and heart. Due to this the heme proteins are among the most important cellular targets of CO. Another potential target of CO is the cytochrome P450 family of enzymes. P450s are involved in oxidative metabolism of drugs and xenobiotics. Physiological levels of CO are usually too low to inhibit P450s, however exogenous exposure to CO can lead to inhibition of P450s isoenzymes such as CYP2D6, CYP3A and CYP2C affecting drug metabolism and vascular tone [77,78]. Ferrous cytochrome c oxidase, which is a key respiratory chain enzyme, can also bind CO, and this may play a biological role under hypoxic conditions [79].

**Table 2:** Selected biological properties of the carbon monoxide versus nitric oxide.

Biological Property	Carbon Monoxide	Nitric Oxide
Main substrates	Heme [4]	l-arginine [4]
Generating enzymes	Heme oxygenases [80]	NO synthases [80]
Inducer	Free radicals [4]	Acetylcholine, endotoxin
Inhibitor	zinc protoporphyrin-IX [4]	NG-nitro-l-arginine methyl ester
air to lung partition coefficient (log P_lung_)	−1.69 [81]	
blood to lung partition coefficient (log K_blood_)	−1.69 [81]	
Protein targets	cGMP, KCa channel [4]	cGMP, KCa channel
Amino acid targets	histidine [4]	cysteine
Production tissue source	endothelial cell < smooth muscle cell [4]	endothelial cell > smooth muscle cell
Production rate in the human body	16.4 μmol/h, 500 μmol/daily [82]	between 0.15 and 2.2 μmol/h, between 4 and 53 μmol/daily [83]
Half-life in solution	Minutes [4]	<1 s [83]
Lethal concentration	1000 ppm [84]	
Metabolism	None [33]	Rapid conversion to nitrite/nitrate [33]
Preclinical efficacy		
Pulmonary hypertension	250 ppm for 1 h/d has long-term efficacy [85]	20–80 ppm has rapid efficacy [66,86]
Sepsis/acute respiratory distress syndrome	250 ppm for 4 h promotes bacteria clearance and decreases inflammation [86]	0.2–20 ppm for 4 h decreases pulmonary hypertension but has no effect on inflammation [87]
Myocardial ischemia	250–1000 ppm for 24 h prevents ischemia reperfusion injury [12]	80 ppm for 60 min prevents ischemia reperfusion injury [88]
pharmacological application	antiinflammatory; protection from tissue reperfusion injury; vasodilatation; antiapoptotic; anti-proliferative; anti-hypoxia; antiaggregatory; anti-ischaemi; [89,90]	antiinflammatory; antiatherogenic; antihyperglycemic; antioxidative; antihypertensive; antimicrobial; antinociception, anticancer; gastric cytoprotection; antithrombogenicity; [74,91]

CO can also bind to and activate sGC similarly but less effectively than NO and induces vasorelaxation in rat-tail arteries [92] and it also inhibits platelet aggregation [93,94].

Some CO is also produced in normal animal metabolism in low quantities (approx. 6–10 cm^3^ per day [89,95]) and is thought to have some normal biological functions. Its endogenous role includes signal transduction in the regulation of nervous and vascular functions and cellular homeostasis. It mediates signalling processes in the brain, liver, and endothelium and inhibits the expression of synergic inflammatory mediators such as TNFα, interleukin family: IL-1, IL-1β, IL-2, IL-6, IL-10, lipopolysaccharide/endotoxin (LPS), prostaglandin-2 (PGE-2), cyclooxygenase 2 (COX-2), intercellular adhesion molecule 1 (ICAM-1), and the receptor activator of nuclear factor κB ligand (RANKL) [90]. The physiological effects of CO depend on its ability to form complexes with the heme moieties of cellular hemoproteins. Due to this, the vital role of CO has been confirmed in organisms lacking HO-1 [96]. CO administered to rodents and large animals at concentrations 10–20-fold below the lethal concentration revealed its remarkable therapeutic value [34]. Notably, this includes antiinflammatory activity as demonstrated in preclinical animal models of inflammation, acute lung injury, sepsis, ischemic/reperfusion injury, and organ transplantation. Additional experimental indications include pulmonary fibrosis, pulmonary hypertension, metabolic diseases, and pre-eclampsia [97].

NO is the most reactive of the physiological gases, having the same effective size and polarity as the O_2_ molecule [30]. In the presence of oxygenated Hb, NO is rapidly metabolized to nitrate with the formation of met-Hb. Met-Hb in erythrocytes is rapidly reduced to ferrous-Hb by met-Hb reductase [86]. Note that NO has a great affinity with blood Hb, far more than O_2_ or even CO. Hb scavenges NO through the high-affinity ferrous binding sites on the heme moiety (Figure 5). Comparison of the Hb binding affinity between NO and oxygen and between NO and CO revealed the following proportions: NO/O_2_ ≈ 8000 [29] and by other authors NO/O_2_ ≈ 1500 [98], while the ratio of NO/CO ≈ 40. The comparison of these values confirms the high reactivity of NO. Additionally, it is believed that NO plays an important role in gastric cytoprotection, possibly by increasing mucosal blood flow, and mucous/fluid secretion by the gastric epithelial cells.

Summing up, besides the concentration, other factors such as the duration of NO and CO exposure and the kinetic parameters of these gasotransmitters play a key role in their biological signaling [77]. Table 2 compares the selected biological properties of the CO versus NO. Both direct and indirect physiological and pathophysiological effects of NO and CO have their origin in overlapping molecular targets including metalloproteins (Table 2). Moreover, their biological interrelationships (Figure 4 and Figure 5) and the similar roles (Figure 2 and Figure 3) make it possible to assume that CO can replace NO under NO deficiency conditions to regulate its bioavailability [92,93,94].

## 5. CO and NO—Gaseous Neurotransmitters

Since the role of NO as a messenger molecule has been established, the cognition of neuronal communication in the CNS has altered, introducing the concept of gaseous transmitters [57]. Regarding the biological importance of gasotransmitters, the most common indices marking their uniqueness are abbreviated by SAVE; where S stands for simplicity of molecular composition and structure, A for availability, due to their presence in all organs, cells, and many intracellular organelles in significant abundance, V is for volatility, reflecting volatility regarding their gaseous nature, and E is for effectiveness, associated with the fact that their extraordinary and widespread cellular effects occur at an extremely low endogenous concentration [99]. Gasotransmitters significantly differ from classical transmitters. It is worth emphasizing that, unlike other signaling molecules interacting with specific receptors where structural features determine biological function, gasotransmitters’ biological activity only depends on their physicochemical properties. The amphiphilic chemical nature that allows the gasotransmitters to diffuse in the cytosol and through lipid membranes is among the most noteworthy features that distinguish them from classical ones [100]. Gases are able to affect a wide range of cells in their vicinity, which resulted in the coining of the term ‘‘volume signaling’’ that reflects this mechanism of action. Table 3 includes the comparison of important features of gasotransmitters (NO and CO) and classical neurotransmitters.

**Figure 6:**
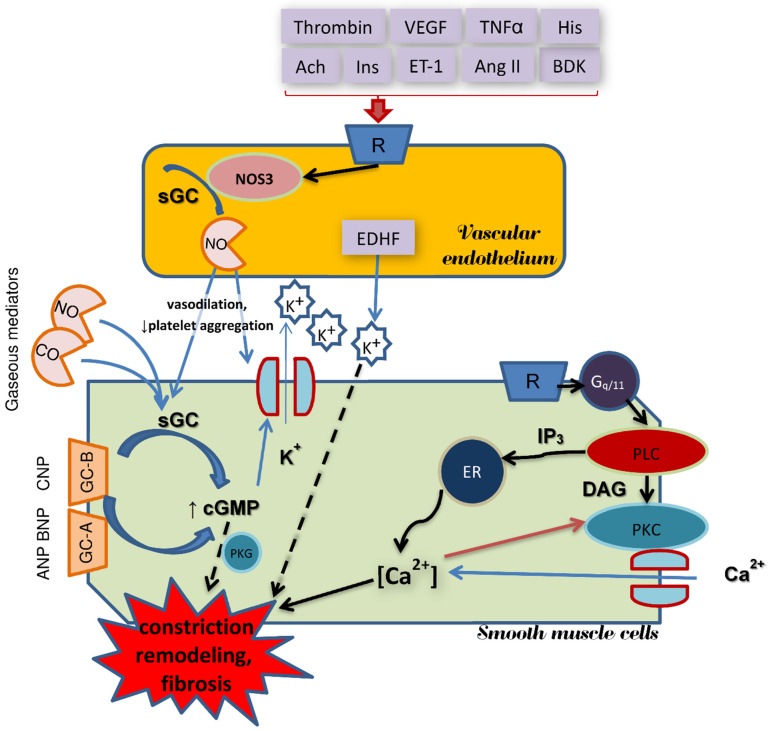
Schematic illustration of gaseous mediators’ intervention in function of vessel wall. Solid lines indicate stimulation processes, dashed lines—inhibition. Natriuretic peptide type A (ANP), B (BNP) and C (CNP); cyclic guanosine monophosphate (cGMP); diacylglycerol (DAC); endoplasmic reticulum (ER); membrane guanylate cyclase type A (GC-A) and B (GC-B); soluble guanylate cyclase (sGC); inositol 1,4,5-trisphosphate (IP3); endothelial nitric oxide synthase (NOS3); protein kinase C (PKC); cGMP dependent protein kinase G (PKG); phospholipase C (PLC); G protein-coupled receptor (R); Gq alpha subunit family (Gq/11); carbon monoxide (CO); nitric oxide (NO); potassium ion (K^+^); calcium ions (Ca^2+^); endothelium-derived hyperpolarizing factor (EDHF); stimuli activating NOS3: thrombin, bradykinin (BDK), vascular endothelial growth factor (VEGF), tumor necrosis factor (TNFα), histamine (His), acetylcholine (Ach), insulin (Ins), endothelin type 1 (ET-1), angiotensin II (Ang II).

The stimulation of the CO and NO pathways, because of their biochemical properties and clinical activity, can be considered as a target for pharmacotherapy. In the pathogenesis of cardiovascular diseases progression such as heart failure, arterial hypertension, and coronary artery disease, among the most important elements is increased contractility, fibrosis and remodeling [101,102,103]. Current intervention, according to the clinical setting, can base on gaseous mediator donors or the activation of the GC pathway. Gaseous mediators, regardless of the source and the route of delivery to the intercellular space, are interesting metabolic interventions directly in disease progression pathways (Figure 6). The results of clinical and experimental trials support the hypothesis that pharmacological intervention at this molecular level is effective and safe [104,105,106,107].

## 6. CO and NO as Potential Therapeutic Agent

According to the literature data on CO and NO (ATC code: V04CX08 [108] for CO; R07AX01 for NO [109]; and DrugBank ID: DB11588 for CO and [110] and DB00435 for NO [111]), there are several NO-related drugs in current clinical use, while CO-based drugs are not yet used in daily clinical therapy. Recent research has demonstrated the role of CO as a gasotransmitter, with critical physiological functions in mammals and biological importance comparable to that of NO [112,113].

### 6.1. CO as a Prodrug

**Figure 7:**
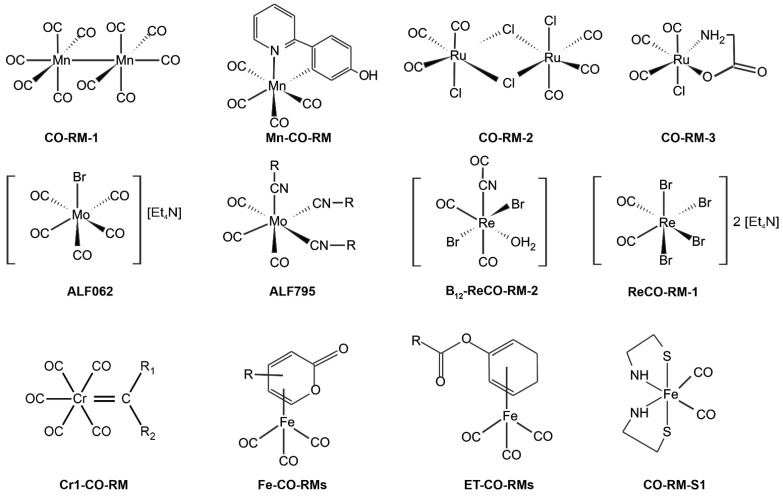
Structures of selected well-known CO-releasing metal complexes [21,120,121,122].

Carbon monoxide, with cytoprotective and homeostatic properties [85], recently has gained recognition as a potential therapeutic agent (Table 2) and has entered multiple clinical trials [114] such as ClinicalTrials.gov (accessed on 26 April 2021) identifier (NCT number): NCT03616002 [115]; NCT03067701 [115]; NCT02530242 [116]; NCT00122694 [117]; NCT04610554 [118]; NCT02425579 [119]. The treatment of several vascular diseases induced the development of new prodrugs, increasing the endogenous production of CO by HO, such as CO-releasing molecules (CORMs, Figure 7) [90,112].

**Figure 8:**
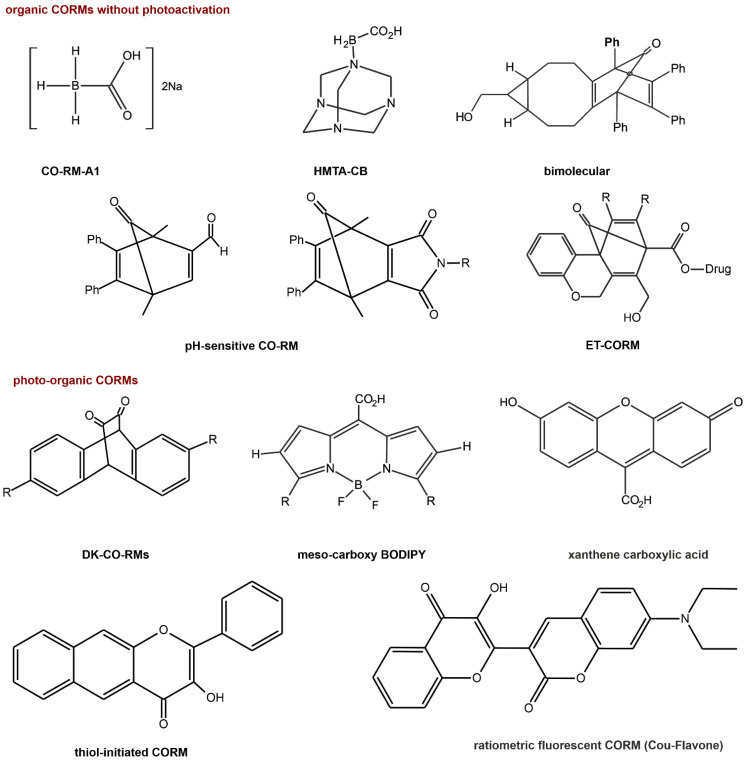
Structures of selected well-known nonmetallic CO-releasing molecules [129,130,131,132].

An alternative to the oral or injection administration of CO is CORM-stable solid complexes of CO [120]. Another classification of this group of compounds is based on the mechanism of CO release activation. This classification includes solvent-triggered CORMs, photo-CORMs, enzyme-triggered CORMs (ET-CORMs), thermal triggered CORMs, oxidation-triggered CORMs, pH-triggered CORMs, etc. [123]. The first generation of CORMs consists of organometallic compounds. They usually contain essential trace elements (especially Fe, Mg, Cr, Co, Mn, and Mo) as well as nonphysiological metals (Ru, Rh, W, Re, and Ir) surrounded by some carbonyl groups (CO) as coordinated ligands (Figure 7) [124,125]. Thus far, few organometallic compounds have been introduced as potential therapeutic agents. The research confirmed the difficulties of the therapeutic application of these compounds resulting from the physicochemical properties of these complexes, such as (i) weak or rapid dissolution in water, (ii) the uncontrolled release of CO (t_1/2_ < 1 min), (iii) the reactivity of metals with biological substances (e.g., nucleophilic and electrophilic chains side proteins), (iv) central metal toxicity [21,126,127]. Therefore, to avoid transition metals, nonmetallic CORMs were developed, becoming second-generation CORMs. As organic CO donors, initially, this class included systems containing boranocarbonates (H_3_BCO_2_), boranocarbamates (H_3_BCO), hexamethylenetetramine carboxyborane (HMTA-CB or HMTA-BH_2_COOH), methylene chloride (MC) [128,129]. However, boranocarboxylate derivatives generate BH_3_ and one equivalent of hydroxides upon CO release [127]; in recent years, other organic forms of CO delivery have been reported (Figure 8).

CORMs have shown a variety of activities, such as vasodilatory, anti-inflammatory, antiapoptotic, anti-ischemic, anticancer, and cardioprotective activities, and have also been proven to regulate mitochondrial activity, respiration experimental colitis, chemically induced liver injury, and ischemia reperfusion kidney injury [133,134,135]. The major advantages of nonmetallic CORMs are potentially low toxicity and property tuning by easy modification [120,136]. Compared to metallic CORMs, which can release multiple COs from each molecule, most anonmetallic CORMs have relatively low CO content and produce organic molecules together with CO [129,130]. Additionally, the organic product could also be a drug that works together with CO to achieve better therapeutic effects [131].

Many studies have demonstrated that CO inhalation at low doses (<250 ppm) offers protection against inflammation and ischemic injury in the heart, liver, and kidney [38,137] NCT03067701 [115]. One benefit of CO over NO is that it is more effective in controlling vessel tone in oxidative stress environments.

### 6.2. NO as a Prodrug

Direct NO gas administration can be used in clinical settings since it can enhance arterial oxygenation in patients with acute respiratory distress syndrome [138]. NO gas administration in newborns treated for pulmonary hypertension is approved by the US Food and Drug Administration (FDA) and European Medicines Agency (EMA), and it can be used as recue treatment in patients with hypoxic COVID-19 symptoms [9,118,139]. 

Drugs that generate NO are known as nitrovasodilators, e.g., organic nitrates (RONO) including nitroglycerin (NTG, glyceryl trinitrate) [140] amyl nitrite, isosorbide mono- and dinitrate (ISMN and ISDN, respectively), erythrityl tetranitrate and inorganic nitrate including sodium nitroprusside (SNP) [141]. These medications have long been used to reduce blood pressure (and other coronary artery diseases) and treat angina pectoris [87]. The NTG is the most notorious and has been clinically used for 150 years to relieve acute attacks of angina pectoris. It is available in several preparations for delivery via several routes: oral tablets, sublingual tablets, buccal tablets, sublingual spray, transdermal ointment, and a transdermal patch; it is also available in intravenous formulations, which are used in hospitalized patients with angina, hypertension, or heart failure [140]. NTG has been FDA-approved since 2000 and is sold under the brand name Nitrostat [142]. NTG converts to NO in the body [143].

A water-soluble sodium salt, SNP, is comprised of Fe^2+^ complexed with NO and five CN^−^ ions. Acting as a prodrug, SNP reacts with sulfhydryl groups on erythrocytes (as well as albumin and other proteins) to produce NO [144]. Upon binding to vascular smooth muscle, NO triggers the intracellular cGMP-mediated activation of PKG (Figure 3) and the subsequent inactivation of myosin light chains, resulting in the relaxation of vascular smooth muscle. The result of this signaling cascade is the peripheral vasodilation of both arteries and veins (with slightly more selectivity for veins) [141]. Among current NO donors, only RONO and SNP are available for clinical use. However, tolerance often develops in patients taking long-term nitrates. On the other hand, the prolonged administration of SNP may lead to the accumulation of cyanide in the body, which leads to serious therapeutic difficulties [76]. This makes it necessary to search for new therapeutics of this type. In addition to RONO, many compounds with various structures can generate NO in vitro or in vivo, and each class of compounds generates NO through a different mechanism, e.g., enzymatic, nonenzymatic, and reductive/oxidative. The chemical and biological activity of the main classes of NO donors, including M-NO complexes, S-nitrosothiols and sydnonimines and others has been studied. In recent years, the combination of NO donors with specific drugs used in various diseases has been of particular interest. Thus, due to the fact that NO has a wide impact on cancer, from the initiation of neoplastic cell transformation to the progression of cancer in the metastatic cascade, recently, the use of NO donors in combination with anti-neoplastic therapies has been strongly supported by numerous preclinical and clinical studies [145]. NO has been shown to participate in a variety of signaling pathways, including Ras, extracellular signal-regulated kinases (ERK), protein kinase B (Akt), cyclin D1/retinoblastoma (Rb), and the mammalian target of rapamycin (mTOR), which are essential for cancer cells [146]. This is especially the case when NO donors have been co-administered with conventional chemotherapeutic drugs because of their synergistic anti-tumor effects. [76]. 

**Figure 9:**
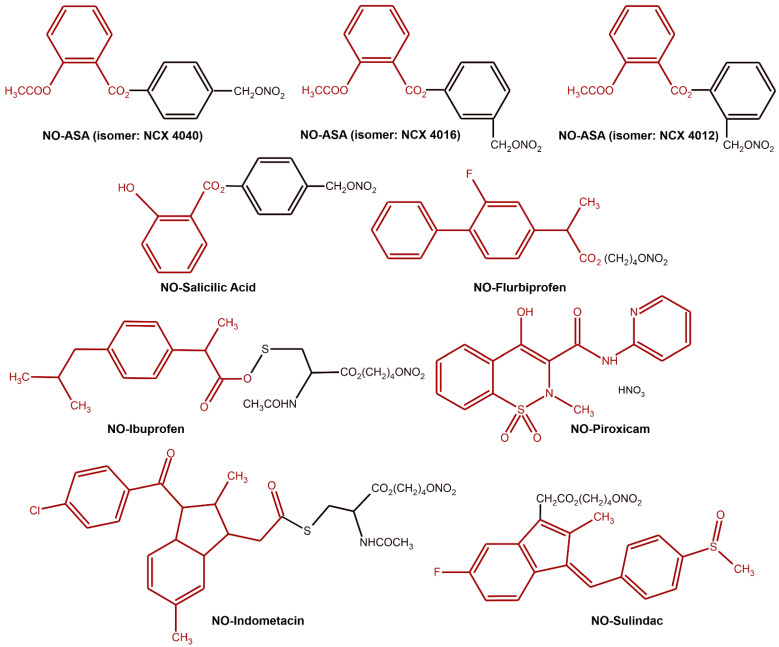
The chemical structure of nitric oxide donors (NO-NSAIDs) [70,150,151]. Classical NSAID is shown in the red colored moieties.

When the body cannot generate sufficient amounts of NO to maintain homeostatic functions, the administration of exogenous NO is a practical method of supplementation, e.g., in patients affected by osteoporosis [147]. As discussed previously, NOS2 and NO are produced as a part of the immune response and have a regulatory role. In various pathological states, e.g., during infection, NO is released by macrophages and other immune cells at concentrations above 1 μM, where it serves as a broad-spectrum biocide [10,148,149]. To effectively exploit these properties of NO, a new class of nitric oxide donating nonsteroidal anti-inflammatory compounds (NO-NSAIDs) was developed (Figure 9). 

NO-NSAID consists of a traditional nonsteroidal anti-inflammatory drug (NSAID) to which a NO-releasing moiety is covalently attached [150]. NO-NASIDs are also known as COX-inhibiting NO donators (CINODs). This group of compounds currently includes NO-aspirin, NO-diclofenac, NO-naproxen, NO-ketoprofen, NO-ibuprofen, NO-flurbiprofen, NO-sulindac, NO-piroxicam and others [152]. Data from several laboratories indicate that NO-NSAIDs may be effective in many diseases, including cardiovascular [153], rheumatological [147,154] and pulmonary diseases [152], Alzheimer’s disease [155] and cancer [150,156]. Numerous in vitro and animal studies have confirmed the enhanced safety profile of NO-NSAIDs in terms of anti-inflammatory efficacy, gastrointestinal and cardiovascular tolerance, and even heart protection compared to their NSAID parents, although there are also reports of severe side effects [157,158,159,160,161]. An excellent example of such a course of research is the research on the hybrid NO and aspirin (ASA) developed by NiCox in the form of three different isomers (NCX 4040, NCX 4016 and NCX 4012 as para-, meta-, and ortoisomers, (Figure 9). NO-ASA has been reported as the most potent (at least 100-fold more active) of the NO-NSAIDs studied to date [151]. Both NCX 4040 and NCX 4012 appear to be 500 to 4,000 times more effective than ASA in inhibiting mammalian cell growth, while the NCX 4016 was rated 100 to 250 times less effective in inhibiting cell growth compared to its counterparts 4040 and 4012, which allows the following ordering of the activities of the individual derivatives NCX 4040 ≈ NCX 4012 > NCX 4016 >>> ASA [162]. Initial phase I clinical trials with NCX 4016 confirmed its better gastrointestinal safety compared to ASA [163], as well as its effectiveness in inhibiting platelet aggregation [164,165,166]. It allowed for the continuation of NCX 4016 studies in phase II in patients with type 2 diabetes and peripheral arterial disease [167,168,169]. However, the clinical development of NCX 4016 was terminated due to the revealed genotoxicity of a potential metabolite of NO-ASA [170]. It is worth emphasizing that the genotoxicity of this metabolite has never been confirmed by others or for that matter for the intact molecule; nevertheless, the development of NCX-4016 as a therapeutic agent was halted [160]. Similarly, NO-naproxen (naproxcinod), which was being evaluated for the treatment of knee and hip osteoarthritis, has completed phase III clinical trials. However, concerns by the FDA about its GI and cardiovascular safety for long-term use did not lead to the approval of NO-naproxen application in 2010 without further studies [171,172,173,174,175]. Therefore, despite the fact that NO-NSAIDs are considered to be a very promising new class of compounds that may affect several areas of modern pharmacology and therapy, final therapeutic decisions should be suspended until the full safety profile of these therapies is known [176].

## 7. Conclusion and Perspective

The importance of NO was discovered in the 1970s, and several years later (in 1987), the physiological role of CO was also proven, leading to a much better understanding of both biochemistry and the development of NO/CO-based pharmaceuticals. Their medical applications are developing rapidly, and it is likely that new drugs based on NO/CO will be used in medical therapies in the near future. However, it seems unquestionable that currently, NO-NSAIDs and CORMs are groups of compounds capable of transporting and releasing controlled NO/CO in cell systems, which constitute a reliable tool for studying the pharmacological effects of these gases or identifying their mechanisms of action. The results of the biology and chemistry of NO-NSAIDs and CORM obtained so far indicate that these compounds are good candidates for the introduction of drugs that release these gases.

There has been significant continuous progress in the area of gasotransmitters since Wang [4] first introduced the idea in 2002. Nowadays, a group of diatomic (NO, CO) and triatomic (H_2_S) small molecules are accepted as gaseous neurotransmitters. The research continues to expand, including in the mediator group more gaseous compounds, such as acetaldehyde (CH_3_CHO), methane (CH_4_), sulfur dioxide (SO_2_), dinitrogen oxide (N_2_O), ammonia (NH_3_) and hydrogen (H_2_) [99,100,177]. Current and pending clinical evaluation will determine the usefulness of these gases as a therapeutic in many different human diseases, especially in the spectrum of diseases of the cardiovascular system.

## Figures and Tables

**Table 1:** Physical properties of carbon monoxide versus nitric oxide.

Physical Property	Carbon Monoxide	Nitric Oxide
Molecular weight	28.01	30.06
Boiling point (°C)	−191.5	−151.8
Melting point (°C)	−205	−163.6
refractive index	1.0003	1.0002697
water solubility	354 mL/dL; 44.3 ppm by mass at STP [30]	67 mg/L [30]
Density (kg/m^3^ vapor)	788.6 [32]	3.027 [32]
Specific gravity (g/L)	1.250 [34]	1.037 [33]
Bond order	2.5 [35]	3 [35]
Bond length, re × 10^−8^ cm	1.151 [35]	1.128 [35]
Reactivity	inert, except binds to hemoproteins; free CO does not readily react with reducing agents, including hydrogen; the coordinated CO has greater reactivity than the free gas, and the reduction of CO can be greatly facilitated by transition metals [36]	highly reactive, very short half life [34]
Vibrational wave number (cm^−1^)	1220 [35]	1180 [35]
Energy activation of oxidation reaction kJ/mol	213 [30]	6.47 [37]
Dissociation energy (kcal/mol)	152.8 [35]	258.9 [35]
Ionization potential (eV)	15.0 [38]	9.27

**Table 3:** Comparison between classical neurotransmitters and gasotransmitters [4,7,56,80].

Feature	Neurotransmitter	Gasotransmitters
Criteria of definition	Complex molecules including different structural groups, e.g., amino acids, peptides, monoamines, purine derivatives, esters. Synthesized in a neuron. Present in the presynaptic terminal and released in amounts sufficient to exert a defined action on the postsynaptic neuron or effector organ. When administered exogenously (as a drug) in reasonable concentrations, it mimics the action of the endogenously released transmitter exactly (e.g., they activate the same ion channels or second messenger’s pathway in the postsynaptic cell). A specific mechanism for removal from their site of action (the synaptic cleft).	Small gaseous molecules. Endogenously and enzymatically generated and their generation is regulated. Well-defined specific functions at physiologically relevant concentrations, e.g., NO and CO both participate in vasorelaxation and synaptic transmission in the CNS. Can freely permeate through a membrane and their effects do not rely on cognate membrane receptors. They can have endocrine, paracrine, and autocrine effects. In their endocrine mode of action, e.g., gasotransmitters can enter the bloodstream, be carried to remote targets by scavengers and released there and can modulate functions of remote target cells. Their cellular effects may or may not be mediated by second messengers but should have specific cellular and molecular targets, e.g., NO and CO activate KCa channels in plasma membrane either directly or mediated by the cGMP pathway.
Examples	Acetylcholine, catecholamines, serotonin, histamine, glutamate, glycine, GABA, ATP and other	NO, CO, and H_2_S
Chemical nature	Lipophilic or lipophobic	Amphiphilic
Action modes:		
Release	Exocytotic vesicle	Cytoplasm release
Re-uptake	+	-
Removal mechanism	Enzyme-dependent	Nonenzymatic: oxidation, scavenging, methylation, etc.
Revert direction	Pre- to postsynaptic membrane (unidirectional)	Bidirectional
Membrane receptors	Necessary	Not necessary

## Abbreviations

Ach	acetylcholine
Akt	protein kinase B
Ang II	angiotensin II
ASA	aspirin
ATC	Anatomical Therapeutic Chemical Classification System
BDK	bradykinin
cAMP	cyclic adenosine monophosphate
Ca^2+^	calcium ions
CH_3_CHO	acetaldehyde
CHEBI	Chemical Entities of Biological Interest
CO	carbon monoxide
CORM	CO releasing molecule
COHb	carboxyhemoglobin
COX-2	cyclooxygenase 2
DAG	diacylglycerol
EDRF	endothelium-derived relaxing factor
EDHF	Endothelium-derived hyperpolarizing factor
ET-1	Endothelin type 1
EMA	European Medicines Agency
ER	endoplasmic reticulum
ERK	extracellular signal-regulated kinases
ERK1/2	extracellular signal-regulated kinases 1/2
FDA	US Food and Drug Administration
FhlA	formate hydrogenlyase transcriptional activator
FAD	flavin adenine dinucleotide
Gq/11	Gq alpha subunit family
G	G protein type G_q/11_
GAF	small-molecule-binding domains of cGMP, cAMP, FhlA
GC	guanylate cyclase
GC-A	membrane guanylate cyclase type A
GC-B	membrane guanylate cyclase type B
sGC	soluble guanylate cyclase
GMP	guanosine monophosphate
cGMP	cyclic guanosine monophosphate
GTP	guanosine triphosphate
H_2_S	hydrogen sulfide
Hb	hemoglobin
His	histamine
HMBD	Human Metabolome Database
HO-x	heme oxygenase type x
HOMO	highest occupied molecular orbital
HPS	heat shock protein
ICAM-1	intercellular adhesion molecule 1
Ins	insulin
IL-x	interleukin type x
IFN-γ	interferons type II
IP_3_	inositol 1,4,5-trisphosphate
ISMN, ISDN	isosorbide mono- and dinitrate
JNK	c-Jun N-terminal kinase
K^+^	potassium ions
K_Ca_	large-conductance potassium channels
KEGG	Kyoto Encyclopedia of Genes and Genomes
L-Arg	L-arginine
LPS	lipopolysaccharide/endotoxin
LUMO	lowest unoccupied molecular orbital
M	metal
metHb	methemoglobin
ANP	natriuretic peptide type A
BNP	natriuretic peptide type B
CNP	natriuretic peptide type C
N_2_O	dinitrogen oxide
NF-κB	nuclear factor kappa-light-chain-enhancer of activated B cells
NADPH	nicotinamide adenine dinucleotide phosphate
NH_3_	ammonia
NO	nitric oxide
NO-NSAIDs	nitric oxide donating non-steroidal antiinflammatory drugs
NSAIDs	non-steroidal antiinflammatory drugs
NOS	nitric oxide synthase
NOS1	neuronal nitric oxide synthase
NOS2	cytokine-inducible nitric oxide synthase
NOS3	endothelial nitric oxide synthase
NTG	glyceryl trinitrate
mTOR	mammalian target of rapamycin
p38 MAPK	p38 mitogen activated protein kinases
PDEx	phosphodiesterase type x
PDB ID	Protein Data Bank Identifier
PGE-2	prostaglandin-2
PKC	protein kinase type C
PKG	cGMP dependent protein kinase G
PLB	phospholamban
PLC	phospholipase type C
R	G protein-coupled receptor
Rb	retinoblastoma
RANKL	the receptor activator of nuclear factor κB ligand
RGS2	regulator of G protein signaling 2
SO_2_	sulfur dioxide
SOMO	single occupied molecular orbital
SNP	sodium nitroprusside
BH_4_	tetrahydrobiopterin
TNFα	tumor necrosis factor α
VASP	vasodilatory-stimulated phosphoprotein
VEGF	Vascular endothelial growth factor
